# Carcinoid Tumor Presenting as Hemoptysis and Elevated Diaphragm

**DOI:** 10.7759/cureus.40586

**Published:** 2023-06-18

**Authors:** Mithil Shah, Brittany Duong, Bernard Karnath, Shawn Nishi, Peter Rasmussen

**Affiliations:** 1 Pulmonary and Critical Care Medicine, University of Texas Medical Branch, Galveston, USA; 2 Critical Care Medicine, University of Texas Medical Branch, Galveston, USA; 3 Internal Medicine, University of Texas Medical Branch, Galveston, USA

**Keywords:** elevated diaphragm, obstructive tumor, endobronchial mass, pulmonary neoplasm, hemoptysis, carcinoid tumor

## Abstract

A bronchial carcinoid tumor is a rare pulmonary neuroendocrine tumor. This report describes a case where a patient experienced multiple episodes of hemoptysis and dyspnea on exertion over the course of five months. An initial chest X-ray showed an elevated right hemidiaphragm with atelectasis, and a follow-up chest computed tomography (CT) scan was ordered to further assess this finding. The CT revealed a tumor occluding 90% of the right main stem bronchus lumen. A bronchoscopy with biopsy was then performed, confirming the diagnosis of a pulmonary carcinoid tumor. The patient underwent surgical resection of the tumor, a right upper lobe sleeve lobectomy, and a mediastinal lymph node dissection, resulting in full eradication of the tumor. This case highlights the need for physicians to maintain a broad differential when evaluating a patient with hemoptysis and an elevated diaphragm.

## Introduction

Lung neuroendocrine tumors, also referred to as bronchial carcinoid tumors, account for 1%-2% of all pulmonary neoplasms and 20%-30% of all carcinoid tumors [1]. Neuroendocrine tumors are commonly found in the gastrointestinal (GI) and respiratory tracts and present as typical or atypical tumors. They are diagnosed by histopathological assessment and typically treated with surgical resection [2]. Individuals with lung neuroendocrine tumors can present with symptoms of an obstructing mass or bleeding, as opposed to signs of serotonin hypersecretion or paraneoplastic syndromes. These patients are usually non-smokers and younger than the typical individual diagnosed with lung cancer. An elevated hemidiaphragm is often caused by phrenic nerve palsy but can indirectly occur from neuronal injury, trauma, or a neoplastic process of the thoracic or abdominopelvic cavities [3]. Our patient had a history of thyroid and prostate cancer. Despite receiving appropriate treatment and disease remission, a history of prior malignancy remains a significant risk factor for future metastases or the development of additional tumors.

## Case presentation

**Figure 1:**
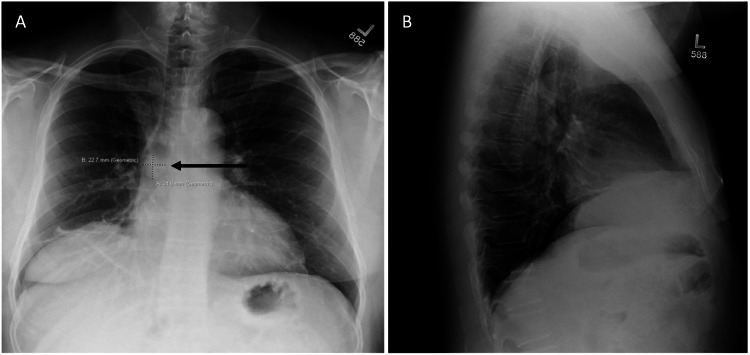
(A) Chest X-ray showing right elevated diaphragm with surrounding atelectasis (black arrow) and the tumor. (B) On the lateral view, the tumor was not clearly evident, so the film was interpreted as not having a tumor.

**Figure 2:**
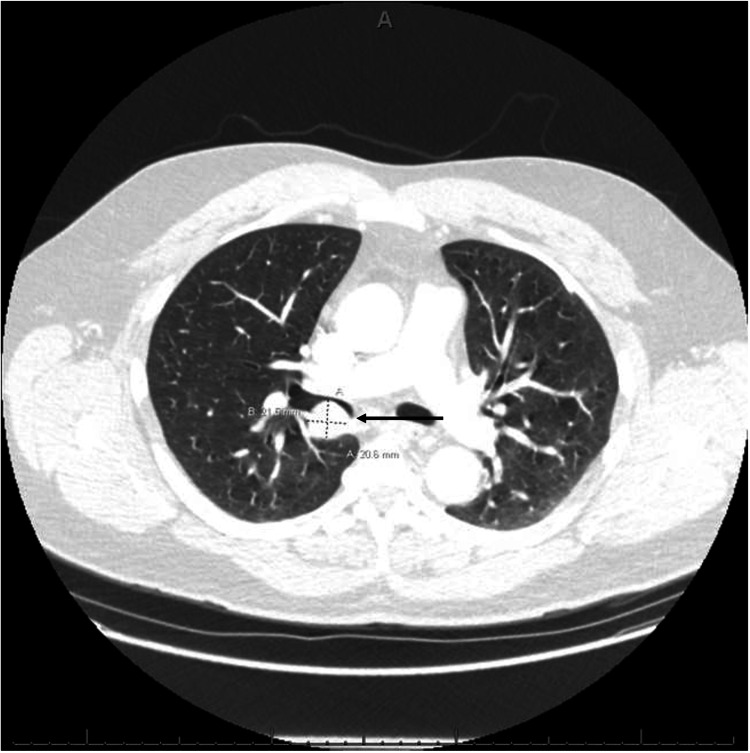
Transverse section of the chest CT showing the endobronchial mass (black arrow). CT: computed tomography

**Figure 3:**
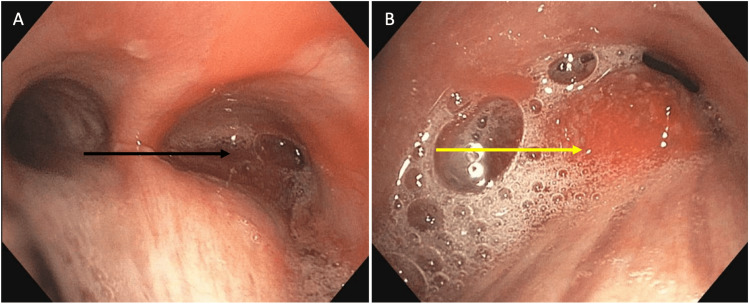
(A) Bronchoscopy in the right main bronchus demonstrated an endobronchial lesion emanating from the posterior membrane, without anterior, lateral, and medial wall involvement (black arrow). The mucosa was highly vascular. (B) Bronchoscopy in the trachea while visualizing the main carina demonstrated that the lumen of the right main bronchus was 90% occluded with pooling secretions (yellow arrow).

Our patient is a 66-year-old male with a history of prostate cancer (status post resection in 2020), Hürthle cell neoplasm of the right lobe of the thyroid (status post resection in 2018), essential hypertension, and type 2 diabetes mellitus who presented to the internal medicine clinic with a five-month history of cough, dyspnea on exertion, and 10 episodes of hemoptysis. Social history was significant for occasional alcohol consumption, but no tobacco or recreational substance use. Physical examination showed an elevated blood pressure of 143/87 mmHg, oral temperature of 36.9°C, pulse of 75 beats per minute, and respiratory rate of 14 breaths per minute. His left lung was clear to auscultation, but wheezing was appreciated over the right lower lobe. There was no clubbing, cyanosis, or lower extremity edema. The right aspect of his thyroid gland was enlarged on palpation. A chest X-ray showed an elevated right hemidiaphragm with surrounding atelectasis (Figure 1). The tumor was not clearly evident on the lateral view and was therefore not included in the initial read. He was then sent for an outpatient chest computed tomography (CT) scan for further evaluation due to persistent symptoms and the elevated right hemidiaphragm. The CT scan showed a 2.4 cm endobronchial mass that extended into the intermediate bronchus 2.2 cm distal to the carina. Multiple areas of severe narrowing and associated post-obstructive sub-segmental atelectasis of the right middle and lower lobes were noted as well (Figure 2). A review of the initial chest X-ray revealed a 2.27 × 2.46 cm nodular density projecting over the mediastinum with adjacent linear opacities in the right middle lobe. During his follow-up office visit, he reported persistent hemoptysis along with purulent sputum. He was then diagnosed with post-obstructive pneumonia and prescribed doxycycline along with a plan to pursue bronchoscopy for tumor biopsy. Two weeks later, the patient underwent endobronchial ultrasound (EBUS) with fine needle aspiration (FNA) of the mass as well as rigid bronchoscopy with laser reduction of the tumor, which was obstructing 90% of the right main stem bronchus lumen. The biopsy was positive for synaptophysin and chromogranin with a 10%-25% cellular proliferation index. One of the lymph nodes was positive for cluster of differentiation 45 (CD45) and was found to have a high lymphocyte proliferation rate. The following month, he underwent video-assisted thoracic surgery (VATS) with right upper lobe sleeve lobectomy and mediastinal lymph node dissection (Figure 3A, 3B). Although lymphocytes in the lymph nodes were positive for CD45, the VATS biopsy was negative for tumor cells in the lymph nodes, and the patient was diagnosed with N0 disease.

## Discussion

Diagnosing pulmonary carcinoid tumors remains a difficult task on account of how rarely they occur. It is important to maintain a broad differential when assessing symptoms such as hemoptysis or dyspnea on exertion. Our patient was correctly diagnosed with a pulmonary carcinoid tumor only after multiple radiological assessments, post-obstructive pneumonia, and finally, a confirmatory biopsy. Given that he was a non-smoker, a neoplastic process was lower on the differential. It is possible that the tumor was too small to be detected by the initial chest X-ray. If the mass was detected on the initial chest X-ray, a contrast-enhanced chest CT scan would have been more promptly obtained [4]. In a smoker, symptoms such as wheezing or dyspnea on exertion could be misdiagnosed as a chronic obstructive pulmonary disease flare or community-acquired pneumonia (CAP), leading to an inappropriate course of steroids or antibiotics. On the other hand, a neoplastic cause might have been higher on the differential for smokers [1]. The fact that the original chest X-ray impression did not detect the mass unfortunately complicated the patient’s course. It was the concern for the elevated hemidiaphragm and persistence of symptoms that led to additional imaging, which then revealed the mass. Physicians should consider an obstructive process when a patient has pneumonia symptoms such as fevers, chills, and a productive cough that persist beyond the typical time frame for a community-acquired infection or fail to improve after antibiotics [1].

Carcinoid syndrome or metastatic disease should be considered in a patient diagnosed with a carcinoid tumor. Both are less common in bronchial carcinoid tumors because they produce less serotonin than midgut carcinoid tumors. Unlike bronchial carcinoid tumors, which are symptomatic when they reach sizes of ≤5 mm, certain gastrointestinal carcinoid tumors can often go undiagnosed until they reach sizes up to 2 cm. At that size, GI tumors often also present with metastatic disease, which is uncommon in bronchial tumors [5]. Regardless, the treatment of choice for carcinoid tumors is surgical resection with adjuvant chemotherapy tailored to each individual case [6]. Some patients with smaller and localized tumors without metastasis might benefit from minimally invasive endobronchial treatment that spares resection of the lung parenchyma [7]. Furthermore, the diagnosis of a typical or atypical tumor as well as the presence or absence of nodal metastasis does not have a significant impact on postoperative survival or tumor recurrence rates [7,8]. Our patient’s tumor obstructed 90% of the right main bronchus lumen. Therefore, he was not a candidate for minimally invasive endobronchial treatment, and surgical intervention was warranted. Patients who undergo carcinoid tumor resection should be monitored for common postoperative complications such as respiratory infections or bleeding. Lastly, one study suggested that recurrence rates can be as low as 3% for typical tumors and as high as 26% in atypical cases [9]. Despite a low risk of recurrence for some patients, lifelong surveillance imaging is necessary given that recurrence can occur as late as 10 years after initial treatment [10].

## Conclusions

This case demonstrates a typical presentation of a low-grade bronchial carcinoid tumor with an appropriate response to surgical resection. The need for primary care physicians to maintain a broad differential diagnosis when treating patients with cough and dyspnea is highlighted by this case. A neoplastic process should be considered when a patient presents with unexplained hemoptysis and dyspnea. Delays in diagnosis or misdiagnosis can prolong the disease course and lead to potentially worse outcomes.
